# Oral Medications for Treating Agitation in a Safety Net Emergency Department

**DOI:** 10.1001/jamanetworkopen.2025.51683

**Published:** 2025-12-30

**Authors:** Jon B. Cole, Kowsar M. Hurreh, Leyla A. Taghizadeh, Andrew P. Laudenbach, Travis D. Olives, James R. Miner, Brian E. Driver

**Affiliations:** 1 Department of Emergency Medicine, Hennepin Healthcare, Minneapolis, Minnesota; 2 Department of Emergency Medicine, University of Minnesota Medical School, Minneapolis

## Abstract

**Importance:**

Best-practice guidelines recommend oral medications for treating agitation in the emergency department (ED). However, there is limited study of their use in the ED, especially in the setting of drug and alcohol intoxication.

**Objective:**

To evaluate the implementation of oral medications to treat agitation in an ED in which intramuscular medications were standard and to increase the use of oral medications to treat agitation.

**Design, Setting, and Participants:**

This quality improvement study analyzed 2 populations. First, all ED patients in an urban level 1 adult and pediatric trauma center from January 1, 2018, to May 31, 2024, were studied to compare the preintervention and postintervention periods. Second, during the 12-month implementation period from September 16, 2020, to August 31, 2021, patients were prospectively observed in an area of the ED dedicated to caring for patients with drug and alcohol intoxication.

**Exposure:**

Oral medications to treat agitation, including olanzapine and lorazepam, were encouraged via education and real-time feedback for ED staff.

**Main Outcomes and Measures:**

The primary outcome was the proportion of patients receiving their first sedating medication via the oral route. Secondary outcomes included data on time to adequate sedation and adverse drug events.

**Results:**

Of 460 600 ED encounters (median patient age, 38 years [IQR, 27-55 years]; 57.8% men; 184 050 [40.0%] before the intervention, 276 550 [60.0%] after), the proportion of patients receiving any sedating medication was similar between time periods (11.8% before intervention vs 11.5% after). The proportion of patients receiving their first sedating medication via the oral route increased after the intervention (7.2% vs 31.4%; difference, 24.2 percentage points [pp] [95% CI, 23.6-24.8 pp]). Among 1178 patients receiving sedating medications during implementation (860 [73.0%] with alcohol intoxication; median breath or blood concentration, 0.22% [IQR, 0.16%-0.28%]), 630 (53.5%) were offered oral medication, of whom 446 (70.8%) accepted it. Time to adequate sedation was 15 minutes (IQR, 7-33 minutes) for oral and 15 minutes (IQR, 9-26 minutes) for intramuscular medications (median difference, 1 minute [95% CI, −0.6 to 2.6 minutes]). There were no significant differences between routes in rates of additional rescue medications (oral, 31.8%; intramuscular, 28.4%; difference, 3.4 pp [95% CI, −2.0 to 8.8 pp]) or adverse drug reactions (oral, 2.7%; intramuscular, 1.1%; difference, 1.6 pp [95% CI, −0.1 to 3.3 pp]).

**Conclusions and Relevance:**

In this quality improvement study, emergency physicians successfully adopted oral medications for treating agitation, primarily from intoxication. No difference was found in time to adequate sedation when oral or intramuscular medications were used as primary therapy. These data support best-practice guidelines that suggest oral medications should be first-line treatment for agitation in the ED.

## Key Points

**Question:**

Can emergency physicians adopt using oral medications as first-line treatment for agitation primarily from intoxication?

**Findings:**

In this quality improvement study evaluating 460 600 ED encounters, the proportion of patients receiving their first sedating medication orally increased from 7% to 31% after 1 year of the intervention, while time to adequate sedation and adverse events, measured prospectively during implementation, were not different between oral and intramuscular routes.

**Meaning:**

The findings suggest that emergency physicians successfully adopted oral medications for treating agitation and that time to sedation and safety were similar regardless of route, supporting use of oral medications as first-line agitation treatments when feasible.

## Teaser

This quality improvement study evaluates outcomes of an emergency department project to implement use of oral medications as first-line treatment for agitation.

## Introduction

Agitation is a common problem in emergency medicine, occurring in up to 2.6% of patients in the emergency department (ED),^1^ and is the cause of up to 1.7 million annual ED visits in the US.^2^ Unlike agitation occurring on psychiatric and inpatient medicine units, agitation in the ED is generally more undifferentiated and more commonly involves intoxication, particularly from alcohol and stimulant-class drugs.^1,3,4^

Verbal de-escalation is the consensus first-line treatment for agitation in the ED.^5,6,7^ However, calming medications, such as antipsychotics and benzodiazepines, are often needed to effectively treat agitation and keep caregivers safe.^1,8^ Many emergency physicians favor the use of intramuscular (IM) medications because of their perceived benefits of reliable drug administration and rapid onset.^9^ Expert guidelines, however, such as Project BETA (Best Practices for Evaluation and Treatment of Agitation), recommend patients should be involved in selecting medications to whatever extent possible, including route of administration.^10,11^ If the patient is able to cooperate with taking oral (per os [PO]) medications, these are preferred over IM medications.^7,11^ When surveyed, patients generally prefer PO medications to IM.^12^

There are few published data on the use of PO medications to treat agitation in the ED. A 2017 scoping review of PO treatments for agitation identified only 6 randomized trials examining 465 patients, with only 2 conducted solely in the ED.^13^ While all 6 trials supported using PO medications, they commonly excluded patients with intoxication,^14,15^ a common cause of agitation in the ED.^3,16,17^ To address this gap in the literature, the 2024 American College of Emergency Physicians Clinical Policy on Patients Presenting With Severe Agitation specifically called for future research examining optimal use of oral and sublingual interventions.^7^

Since the 1980s, our ED has used predominantly IM medications to treat agitation, due to the commonly held belief that ED patients with agitation, who are often intoxicated, are not capable of safely cooperating with PO medication administration.^18^ In spring 2020, departmental leadership approved a plan to educate emergency physicians on the effectiveness of PO medications for agitation. Emergency physicians were then encouraged to offer PO medications for treating agitation when medications were deemed necessary and the PO route was deemed safe. PO medications were encouraged because they are less invasive, may aid in de-escalation, and are in accordance with best-practice guidelines.^11^ This analysis presents the results of a quality improvement project to implement the use of PO medications to treat agitation in our ED.

## Methods

### Study Design and Setting

This quality improvement study was conducted in the ED of an urban, county, safety net, level 1 adult and pediatric designated trauma center in Minneapolis, Minnesota, with an annual census of approximately 100 000 patients and an emergency medicine residency that has been present since 1972. Minnesota is an upper midwestern state with a disproportionately high rate of binge drinking.^19,20^ Public intoxication in Minnesota is not a crime,^21^ and law enforcement frequently transports intoxicated individuals to hospitals in lieu of jail.^22^ The study conformed to the Standards for Quality Improvement Reporting Excellence (SQUIRE) reporting guideline for quality improvement studies.^23^ The quality improvement project was approved by the ED chair of Hennepin Healthcare using institutional guidelines.^24^ The Hennepin Healthcare institutional review board approved analysis of all ED patients from January 1, 2018, to May 31, 2024, to assess the outcomes of the intervention and waived informed consent because the research met minimal-risk criteria, the intervention was incorporated into routine clinical care for all patients, and the study could not practicably be conducted otherwise.

We evaluated 2 complementary populations: (1) all ED encounters before and after the intervention (2018-2024), as described subsequently, and (2) a prospective cohort of patients with agitation observed during the active implementation period (2020-2021). The quality improvement intervention described herein occurred throughout the entire ED. Additional detailed data collection occurred in a dedicated 16-bed locked intoxication observation unit inside this ED that cares for approximately 7000 patients per year, which was created after closure of county-run detoxification facilities in the 1990s.^17,25,26,27,28^ Emergency medicine physicians and nurses care for patients here just as they would in other ED units. The baseline rate of PO sedating medications for agitation in this unit was 2%.^17^

### Quality Improvement Intervention

Beginning on July 7, 2020, best-practice guidelines and primary literature on the use of PO medications for treating agitation were presented in a series of lectures to all ED staff. Clinicians were encouraged to use PO rather than IM medications across the entire ED to treat agitation, when feasible. A permanent order panel was built in the electronic medical record to facilitate ordering PO olanzapine and lorazepam, and the orally disintegrating tablet preparations of olanzapine and risperidone were stocked in the ED. Treating physicians always determined drug, dose, and route for individual patients.

The active intervention period ran from September 16, 2020, to August 31, 2021, during which trained independent staff were stationed within the locked observation unit to identify eligible patients and to perform data collection. These staff were independent from the care teams and specifically trained for observational data collection, as described elsewhere.^17,29^

Real-time data from the locked unit were used to give ongoing feedback to clinicians and were presented in faculty meetings throughout the intervention period to reinforce and expand the use of PO sedating medications throughout the ED. Although prospective data collection was limited to the locked intoxication unit for feasibility reasons, given its high volume of agitated patients, findings were used iteratively to inform practice throughout the entire department.

### Selection of Participants

#### All ED Encounters

Study population 1, the retrospective population, included all ED encounters between January 1, 2018, and May 31, 2024, including patients before, during, and after the intervention. These data were used to evaluate changes in oral sedating medication use over time. Demographic and encounter-level data were obtained from the electronic health record.

#### Patients With Agitation During the Active Intervention Period

Study population 2, the prospective study population, included patients with agitation cared for in the locked observation unit during the active intervention period (September 16, 2020, to August 31, 2021). Agitation in this population was defined as a score of 1 or higher on the Altered Mental Status Scale (AMSS), an ordinal,^30^ validated^31^ agitation scale with scores ranging from −4 (unresponsive) to 0 (normal) to 4 (most agitated). The AMSS is used clinically in a fashion similar to the Richmond Agitation-Sedation Scale.^32^ A change in AMSS score has been used as the outcome measure in multiple clinical trials examining agitation^33,34,35,36^ and correlates strongly with the Behavioral Activity Rating Scale, another validated agitation scale (eTable 1 in Supplement 1).^36^ This population was used to describe the outcomes of PO medications for treatment of agitation in the ED.

### Methods of Measurement

#### Study Population 1

For the first study population, a hospital data analyst extracted data from the electronic medical record. Variables included patient demographics, mode of arrival, alcohol concentration (if measured), disposition, diagnosis, and details of sedating medication administration. Race and ethnicity, self-reported by the patient during routine clinical care and documented in the electronic medical record, were included to describe the study population; no analyses were performed based on these variables. Categories as described in previous work^19^ were American Indian or Alaska Native, Asian, non-Hispanic Black (hereafter, *Black*), Hispanic, Native Hawaiian or Other Pacific Islander, and non-Hispanic White (hereafter, *White*). Diagnoses of interest included agitation, alcohol intoxication, drug intoxication, and bipolar disorder or psychosis (diagnostic codes are in eTable 2 in Supplement 1). Sedating medications included olanzapine, droperidol, haloperidol, risperidone, lorazepam, and midazolam, the most common agitation treatments used in our ED.

#### Study Population 2

For the second study population, trained staff were present in the locked unit 24 hours per day and observed patients throughout their entire stay. Detailed data collection commenced if agitation (AMSS score ≥1) was present at any point during the ED encounter. Staff prospectively recorded patient demographics; vital signs; alcohol concentration (if measured); degree of agitation (AMSS score); risk of patient violence as measured by the MIAHTAPS violence risk assessment tool evaluating altered mental status, irritability, agitation, history of violence against others, threats (verbal or physical), attacking objects, and pacing and/or staring (eTable 3 in Supplement 1)^37^; whether and how de-escalation was performed by a health care worker^5^; whether a PO sedating medication was offered and the patient’s response; details of sedating medication administration, including medication, route, and timing; time from medication administration to adequate sedation (defined as AMSS score ≤0); need for additional sedating medication during the ED encounter; whether the patient displayed verbal abuse, threat of violence, or a violent act^29^; the use of restraints^19^; ED length of stay; ED disposition; and whether an adverse drug reaction occurred. Adverse drug reactions included hypotension (systolic blood pressure <90 mm Hg), bradycardia (heart rate <60 beats/min), hypoxemia (oxygen saturation <90%), and tracheal intubation.

### Outcomes

The primary outcome in the first (retrospective) population of all ED patients was the proportion of patients receiving sedating medications who received an oral sedating drug as the first medication given. This outcome was selected to monitor the magnitude and durability of changes made regarding the use of PO sedating medications during and after the intervention. For the second (prospective) population, exploratory outcomes included time to adequate sedation, need for additional sedating medications, and adverse drug reactions.

### Statistical Analysis

The first analysis included all ED encounters from January 1, 2018, through May 31, 2024, stratified by whether patients were cared for before or after the intervention. Data are presented as counts and proportions or medians with IQRs, as appropriate.

We applied interrupted time series analysis (ITSA) to assess changes in the monthly proportion of patients receiving oral sedating medications in the ED. ITSA, a segmented ordinary least squares regression method for time series data, identifies significant shifts in the level (intercept) and trend (slope) of outcomes following an intervention.^38^ This approach is advantageous as it accounts for secular trends inherent in population-level data, unlike basic pre-post tests. Adjustments for individual characteristics are typically not needed unless potential confounders change simultaneously with the intervention. Using ITSA, we estimated the association of the quality improvement intervention with the rate of use of an oral sedating medication and report estimated postintervention changes in intercept and slope. The model adjusted SEs for autocorrelation.

The second analysis included patients with agitation prospectively observed in the locked intoxication observation unit during the active intervention period (September 16, 2020, through August 31, 2021). Patients were categorized by their initial sedation strategy—no sedation, oral (PO) sedation, or parenteral sedation as the first sedating medication. Data are presented as counts and proportions or medians with IQRs.

Time to adequate sedation and need for additional sedating medications were compared by calculating the difference in the proportions or median difference, as appropriate, between groups and the associated 95% CI. Hodges-Lehmann median between-group differences and the associated 95% CIs were calculated for continuous variables.

Analyses were performed using Stata, version 15.0 (StataCorp LLC). Time-series regression analyses were conducted using Stata’s time-series suite and median differences were estimated using the cendif package. All other analyses were descriptive or tabular. Statistical significance was assessed by examining whether 95% CIs for between-group differences excluded 0.

## Results

### Analysis of ED Patients From 2018 to 2024

The first analysis included 460 600 ED encounters from January 1, 2018, through May 31, 2024 (median patient age, 38 years [IQR, 27-55 years]; 57.8% men, 42.2% women), including 184 050 (40.0%) before the intervention and 276 550 (60.0%) after. Across all encounters, 6.5% of patients were American Indian or Alaska Native; 2.1%, Asian; 44.8%, Black; 14.3%, Hispanic; 0.2%, Native Hawaiian or Other Pacific Islander; 30.0%, White; and 2.1% had unknown or undisclosed race and ethnicity. Characteristics of patients were similar in both time periods (Table 1). Overall, 66 436 patients (14.4%) were cared for in a locked unit.

**Table 1.** Patient Characteristics and Sedating Medication Details for All ED Patients From 2018 to 2024^a^

Characteristic	Patients, No. (%)	
	Preintervention (n = 184 050)	Postintervention (n = 276 550)
Age, median (IQR), y	38 (27-54)	39 (27-55)
Sex		
Men	105 663 (57.4)	160 549 (58.1)
Women	78 387 (42.6)	116 001 (41.9)
Race and ethnicity^b^		
American Indian or Alaska Native	12 738 (6.9)	17 280 (6.2)
Asian	3822 (2.1)	5851 (2.1)
Black, non-Hispanic	82 493 (44.8)	123 908 (44.8)
Hispanic	21 674 (11.8)	44 389 (16.1)
Native Hawaiian or Other Pacific Islander	363 (0.2)	646 (0.2)
White, non-Hispanic	59 340 (32.2)	78 807 (28.5)
Unknown or patient declined	3983 (2.2)	5669 (2.1)
Mode of arrival		
Emergency medical services	60 493 (32.9)	102 876 (37.2)
Police	4995 (2.7)	4975 (1.8)
Walk-in	118 562 (64.4)	168 699 (61.0)
Cared for in locked unit^c^	25 138 (13.7)	41 298 (14.9)
Breath or blood alcohol concentration		
0^d^	20 843 (11.3) [26 081]	24 663 (8.9) [37 319]
If >0, median (IQR), %	0.20 (0.14-0.27)	0.21 (0.14-0.27)
Disposition		
Discharged	144 019 (78.2)	217 847 (78.8)
Admitted to the hospital	37 996 (20.6)	50 760 (18.4)
Left before discharge	2035 (1.1)	7943 (2.9)
Diagnosis^e^		
Agitation^f^	3512 (1.9)	2573 (0.9)
Alcohol intoxication	30 512 (16.6)	43 008 (15.6)
Drug intoxication	2890 (1.6)	5815 (2.1)
Bipolar or psychosis	1420 (0.8)	2373 (0.9)
Sedating medication received, any^g^	21 711 (11.8)	31 934 (11.5)
Route of first sedating medication, No./total No. (%)		
Intramuscular or intravenous	20 137/21 711 (92.8)	21 902/31 934 (68.6)
Intramuscular	8583/21 711 (39.5)	5532/31 934 (17.3)
Intravenous	11 554/21 711 (53.2)	16 370/31 934 (51.3)
Oral	1574/21 711 (7.2)	10 032/31 934 (31.4)

Abbreviation: ED, emergency department.

^a^ Table includes all ED encounters from January 1, 2018, through May 31, 2024.

^b^ More than 1 category was listed for some patients; thus, the sum exceeds the total number of patients.

^c^ Refers to patients cared for in the dedicated locked 16-bed intoxication observation unit inside the ED, as described in the Methods.

^d^ Numbers in brackets indicate how many encounters had breath alcohol measured.

^e^ Diagnosis definitions are in eTable 2 in Supplement 1.

^f^ Not every patient treated for agitation received a diagnosis of agitation.

^g^ Included the following medications by any route: olanzapine, droperidol, lorazepam, midazolam, haloperidol, and risperidone.

The proportion of patients receiving any sedating medication was similar between time periods (11.8% before intervention vs 11.5% after). Among patients who received a sedating medication, the proportion receiving an IM injection decreased after the intervention (39.5% vs 17.3%; difference, −22.2 percentage points [pp]; 95% CI, −23.0 to −21.4 pp) and the proportion receiving a PO sedating medication increased (7.2% vs 31.4%; difference, 24.2 pp; 95% CI, 23.6-24.8 pp). Findings were more pronounced among the 89 244 patients (19.4%) at risk for agitation (ie, those diagnosed with drug or alcohol intoxication, bipolar mood disorder, or psychosis and those cared for in a locked unit) (eTable 4 in Supplement 1).

**Figure 1.**
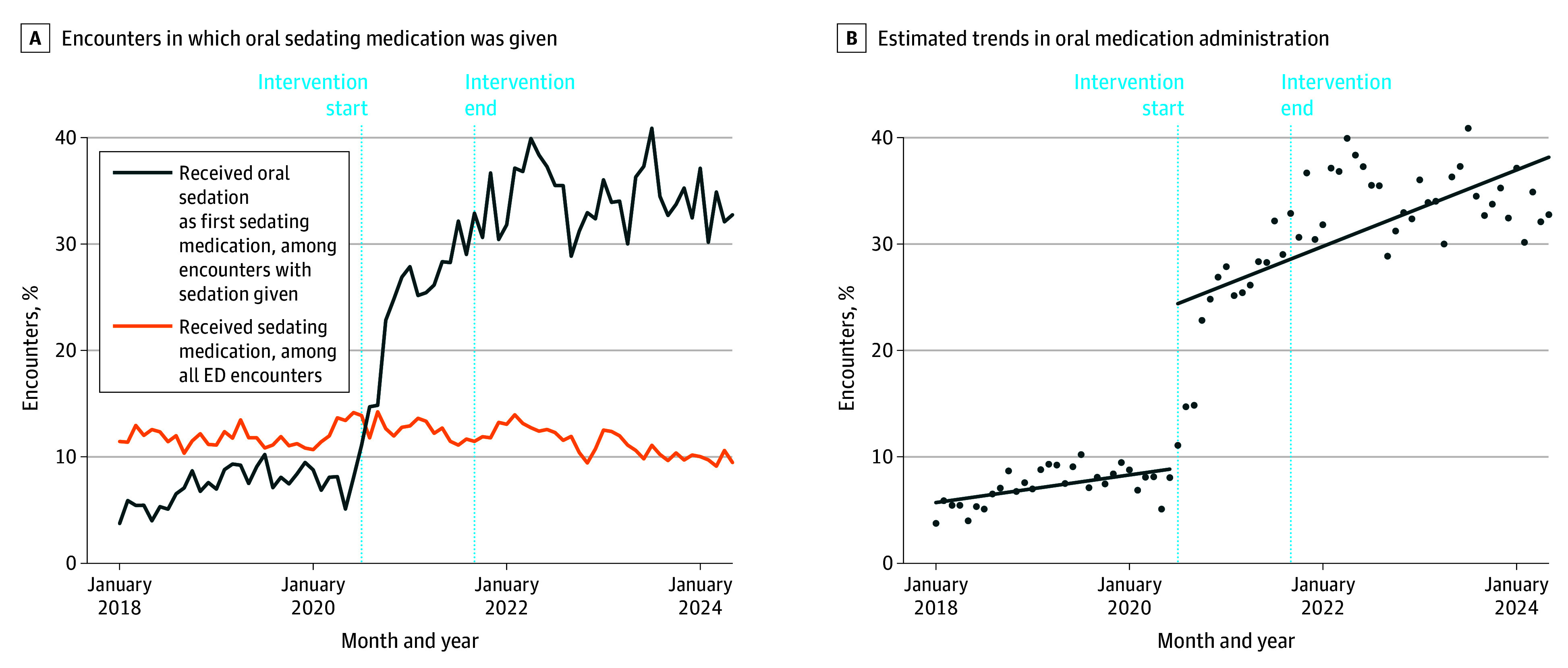
Use of Oral and Intramuscular Sedating Medications Over Time Sedating medications included droperidol, haloperidol, olanzapine, risperidone, midazolam, and lorazepam. The quality improvement intervention started with education of the emergency department (ED) staff on July 7, 2020, and ended August 31, 2021 (dashed vertical lines). B, Fit lines represent the estimated trends for oral medication administration before and after the intervention.

The monthly proportion of patients receiving a PO sedating medication is shown in Figure 1A. Interrupted time series analysis revealed a postintervention change in intercept of 15.4% (95% CI, 10.7%-20.2%), indicating an increase in the proportion receiving an oral sedating medication at the start of the intervention among those receiving any sedating medication. The change in slope was 0.2% (95% CI, 0.02%-0.4%), indicating that the use of PO medications persisted and continued to increase after the intervention, compared with the preintervention trend (Figure 1B).

### Analysis of Patients With Agitation Observed Prospectively During the Intervention

The second analysis included patients with agitation prospectively observed in the locked intoxication observation unit during the intervention period (September 16, 2020, to August 31, 2021). Of 7493 patients screened, 1686 patients (22.5%) had agitation (AMSS score ≥1) and were included. Among patients with agitation, 508 (30.1%) did not receive sedating medications, 446 (26.5%) received a PO sedating medication, and 732 (43.4%) received an IM sedating medication. Most patients with agitation (1262 [74.9%]) had a detectable breath or blood alcohol concentration (median concentration, 0.22%; IQR, 0.16%-0.28%; range, 0.01%-0.48%), and 1362 (80.8%) arrived by ambulance. De-escalation by health care workers was attempted in 1601 patients with agitation (95.0%), usually with multiple different de-escalation strategies (Table 2 and eTable 6 in Supplement 1). Additional patient characteristics are shown in Table 2 and eTable 5 in Supplement 1.

**Table 2.** Characteristics of Patients With Agitation During the Prospective Quality Improvement Intervention

Characteristic	Patients, No. (%) (N = 1686)		
	No sedation (n = 508)	Oral medication (n = 446)^a^	Intramuscular medication (n = 732)^a^
Age, median (IQR), y	41 (32-56)	38 (31-52)	39 (30-51)
Sex			
Men	387 (76.2)	307 (68.8)	498 (68.0)
Women	121 (23.8)	139 (31.2)	234 (32.0)
Race and ethnicity^b^			
American Indian or Alaska Native	84 (16.5)	68 (15.2)	100 (13.7)
Asian	2 (0.4)	5 (1.1)	11 (1.5)
Black, non-Hispanic	245 (48.2)	183 (41.0)	364 (49.7)
Hispanic	13 (2.6)	17 (3.8)	27 (3.7)
Native Hawaiian or Other Pacific Islander	3 (0.6)	0	3 (0.4)
White, non-Hispanic	161 (31.7)	174 (39.0)	229 (31.3)
Unknown	23 (4.5)	15 (3.4)	32 (4.4)
BMI, median (IQR)	27 (23-31)	23 (21-26)	26 (23-30)
Breath or blood alcohol concentration			
>0	402 (79.1)	314 (70.4)	546 (74.6)
If >0, median (IQR), %	0.22 (0.04-0.28)	0.24 (0.18-0.30)	0.21 (0.15-0.27)
Mode of arrival			
Emergency medical services	428 (84.3)	346 (77.6)	588 (80.3)
Police	37 (7.3)	31 (7.0)	69 (9.4)
Walk-in	38 (7.5)	58 (13.0)	56 (7.7)
Other	5 (1.0)	11 (2.5)	19 (2.6)
AMSS score^c^			
On arrival, median (IQR)	1 (1-2)	1 (1-2)	1 (1-3)
≥2 At any point before medication administration	NA	206 (46.2)	572 (78.1)
MIAHTAPS score, median (IQR)^d^	3 (2-4)	3 (2-4)	3 (3-6)
De-escalation attempted for agitation^e^	493 (97.0)	438 (98.2)	670 (91.5)
De-escalation techniques used per patient, median (IQR), No.^f^	4 (2-6)	4 (2-6)	3 (2-5)

Abbreviations: AMSS, Altered Mental Status Scale; BMI, body mass index (calculated as weight in kilograms divided by height in meters squared); NA, not applicable.

^a^ Received as the first sedating medication.

^b^ More than 1 category was listed for some patients; thus, the sum exceeds the total number of patients.

^c^ The AMSS is an ordinal agitation scale with scores ranging from −4 (unresponsive) to 0 (normal) to 4 (most agitated).

^d^ MIAHTAPS is a violence risk assessment tool used at our institution to evaluate altered mental status, irritability, agitation, history of violence against others, threats (verbal or physical), attacking objects, and pacing and/or staring, with scores ranging from 0 (lowest risk) to 12 (highest risk).

^e^ Some patients had more than 1 reason that de-escalation was not attempted.

^f^ De-escalation techniques included offering nourishment, offering an object of comfort, a period of intent listening, identifying the patient’s needs, offering choices or options, calmly encouraging behavior modification, offering support, validating the situation, physical barrier or movement, step-by-step guidance, offering to have the patient use the restroom, or changing the conversation. More details are shown in eTables 5-7 in Supplement 1.

Among the 1178 patients who received either a PO or IM sedating medication (860 [73.0%] of whom had alcohol intoxication), 630 (53.5%) were offered a PO medication, with 446 of those (70.8%) accepting it. Among the 548 patients with agitation who received IM medication without being offered PO medication (46.5%), common reasons that PO medications were not offered included active violence (205 patients [37.4%]) and delirium or confusion (169 patients [30.8%]) (Table 3 and eTable 7 in Supplement 1). PO medications were more successfully implemented in patients with less severe agitation (AMSS score of 1 or 2; 365 of 771 patients [47.3%] received oral medication) compared with more severe agitation (AMSS score of 3 or 4; 30 of 309 patients [9.7%] received oral medication) (eTable 7 in Supplement 1).

**Table 3.** Medication Details Among Patients With Agitation Who Received a Sedating Medication During the Prospective Quality Improvement Intervention

Detail	Patients, No. (%)	
	Oral medication (n = 446)^a^	Intramuscular medication (n = 732)^a^
Offered an oral sedating medication	446 (100)	184 (25.1)
Reason oral medication was not offered, No./total No. (%)		
Active violence	NA	205/548 (37.4)
Delirium or confusion	NA	169/548 (30.8)
Other or unknown	NA	174/548 (31.8)
Medication administration		
Time from ED arrival to sedation administration, median (IQR), min	33 (18-67)	23 (12-55)
Medication given		
Olanzapine^b^	146 (32.7)	564 (77.0)
Olanzapine plus lorazepam	263 (59.0)	0
Droperidol	0	129 (17.6)
Other^c^	37 (8.3)	39 (5.3)
Outcomes		
Time to adequate sedation, median (IQR), min^d^	15 (7-33)	15 (9-26)
Need for additional sedating medication	142 (31.8)	208 (28.4)
Elapsed time from initial to additional sedating medication, median (IQR), min	55 (31-97)	54 (30-122)
Violence or abuse toward health care workers^e^		
Violent act, attempted or inflicted	16 (3.6)	104 (14.2)
Threat of bodily harm	60 (13.5)	213 (29.1)
Verbal abuse	119 (26.7)	351 (48.0)
Physical restraints applied	186 (41.7)	624 (85.2)
Adverse drug reaction^f^	12 (2.7)	8 (1.1)
Hypotension (systolic blood pressure <90 mm Hg)	3 (0.7)	4 (0.5)
Bradycardia (heart rate <60 bpm)	2 (0.4)	3 (0.4)
Oxygen saturation <90%	5 (1.1)	11 (1.5)

Abbreviations: ED, emergency department; NA, not applicable.

^a^ Received as the first sedating medication.

^b^ The only preparation of oral olanzapine stocked in the emergency department during the study period was the orally disintegrating tablet.

^c^ Included lorazepam, midazolam, risperidone, haloperidol, and ziprasidone (eTable 7 in Supplement 1).

^d^ Defined as an Altered Mental Status Scale agitation score of 0 (normal) or lower on an ordinal scale from −4 (unresponsive) to 4 (most agitated).

^e^ These events were observed prospectively by trained nonclinical staff. A violent act was defined as attempted or inflicted bodily harm. Threat of bodily harm was a verbal threat made toward a health care worker. Verbal abuse was defined as the use of harsh or insulting language toward a health care worker.

^f^ Only adverse drug reactions occurring after administration of a sedating medication are included, as determined by the treating physician at the conclusion of the ED visit.

**Figure 2.**
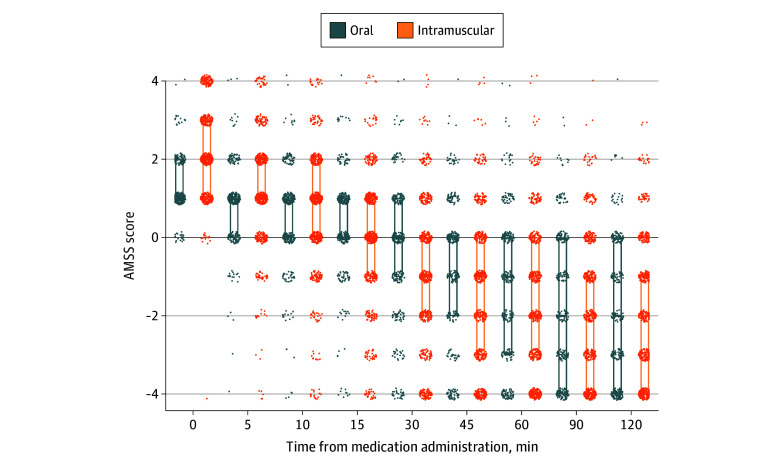
Scatterplot of Altered Mental Status Scale (AMSS) Values Over Time After Medication Administration Axis jittering was performed to improve visualization of individual patient-level data. Each box depicts the interquartile range and median score; when the box spans just 2 scores, the denser cluster is the median and either the upper or lower quartile.

Among the 446 patients receiving oral medication, the most common PO regimens included olanzapine alone, given to 146 patients (32.7%), and olanzapine coadministered with lorazepam, given to 263 patients (59.0%). The most common IM medications included olanzapine, given to 564 of the 732 patients receiving IM medications (77.0%), and droperidol, given to 129 patients (17.6%). The median time until adequate sedation was 15 minutes (IQR, 7-33 minutes) for PO medications and 15 minutes (IQR, 9-26 minutes) for IM medications, with a median difference of 1 minute (95% CI, −0.6 to 2.6 minutes) (Figure 2). Median times to adequate sedation by specific medications ranged from 14 to 16 minutes (eTables 8-9 in Supplement 1). Additional sedation was given to 142 patients (31.8%) who initially received a PO sedating medication and to 208 patients (28.4%) who initially received an IM sedating medication, with an absolute difference of 3.4 pp (95% CI, −2.0 to 8.8 pp). Medications used and outcomes were largely similar to the main analysis in the subgroup of patients who had an AMSS value of 2 or higher at any time before medication administration (eTable 9 in Supplement 1).

Additional details on medication administration, violent events, and restraints are shown in Table 3 and eTable 7 in Supplement 1. There were no significant differences between sedating medication routes in rates of adverse drug reactions (PO, 2.7%; IM, 1.1%; difference, 1.6 pp [95% CI, −0.1 to 3.3 pp]) (Table 3).

## Discussion

We found that emergency physicians quickly and successfully adopted the use of PO medications to treat agitation, occurring particularly in patients with drug and alcohol intoxication, after a focused quality improvement intervention and that this practice persisted for years. The increase in PO medication use was accompanied by a commensurate decline in the use of IM medications, suggesting emergency physicians successfully replaced IM injections with less invasive PO medications. Furthermore, time to adequate sedation and safety outcomes were not different between patients receiving PO or IM medications. Our data support existing best-practice guidelines that, when feasible, PO medications should be first-line treatment for agitation in the ED.^11,39,40^

We found no difference in times to adequate sedation between medication regimens during the quality improvement intervention, consistent with previous randomized trials demonstrating a PO second-generation antipsychotic, with or without coadministered lorazepam, was as effective as an injectable antipsychotic.^14,15,41,42^ However, these trials were small, were frequently conducted outside EDs, and generally examined nonintoxicated patients who could provide informed consent, while the present study’s data examined common, routine ED patients, most of whom had intoxication. Furthermore, our sample size was larger than that of 6 randomized trials on this topic in a scoping review combined,^13^ adding to the generalizability of our data. When considering the pharmacokinetics of the most common treatment in our study, olanzapine, the finding of no difference in time to adequate sedation between PO and IM routes may at first seem unexpected. The time to peak plasma concentration for IM olanzapine is 15 to 45 minutes^43^ but is 6 hours when administered orally.^44^ However, we found olanzapine by either route provided adequate sedation, typically within 15 minutes. The reasons for this remain speculative. It is possible that early absorption of PO olanzapine, while not yet at peak concentration, is sufficient to calm most patients in 15 minutes. It is also possible that inherent de-escalation occurs when the PO route is successfully undertaken, further facilitating olanzapine’s effectiveness, as it preserves patient autonomy by allowing for more active participation in care. As the ideal goal of sedation is calming without oversedation,^11,39,45^ preservation of autonomy may further aid the patient in regaining control.

Previous clinical trials examining medications for agitation conducted in the ED involved either the PO route administered to nonintoxicated, consentable patients^42,46^ or parenteral medications administered to patients (many of whom were intoxicated) under a waiver of informed consent.^47,48,49,50^ Our study helps fill the gap in the literature between these 2 populations. Most of the patients with agitation in our study were intoxicated, similar to the populations from trials with a waiver of informed consent examining parenteral medications, yet over one-third of our study’s patients who received any sedating medication safely took oral medication with excellent outcomes that were similar to previous randomized trials.^33,34,36,49^ While a randomized trial of IM vs PO olanzapine for agitation from intoxication would be ideal methodologically, such a study may not be feasible in the US due to the challenges of informed consent.^51,52,53^

We believe our data will aid other EDs that currently use predominantly IM medications, as implementation data on the use of PO medications are currently sparse.^54,55^ Internally, the iterative use of these data helped spread the use of PO medications for treating agitation to other areas of the ED (Figure 1) and our emergency medical services system.^56^ While the use of PO medications increased substantially after the intervention, IM medications were still often necessary, much as they were in Australian data examining similar populations.^4,57^ Notably, once patients demonstrated any element of physical violence, successful administration of PO medications was less common (eTable 7 in Supplement 1). Future work should focus on identifying tactics to increase the use of PO medications for agitation given their potential safety and effectiveness and less invasive nature.

### Limitations

This study has several limitations. First, the findings from this urban safety net ED may not generalize to other settings. Second, outcomes were assessed in patients who mainly had drug or alcohol intoxication. Results may differ in agitation from other causes. This limitation may be tempered by the sustained adoption of PO sedating medications throughout the ED for all patients. It is unlikely clinicians would have sustained this change if they believed it was ineffective. Third, this was not a randomized trial. The PO and IM groups are likely inherently different. Nonetheless, our data suggest that there is a subset of intoxicated and agitated ED patients who can safely receive PO medications. Fourth, we did not measure satisfaction regarding the use of PO medications in patients or caregivers, though the fact that we observed fewer violent acts against health care workers in patients that accepted PO medications suggests both patients and caregivers found this route to be acceptable. Fifth, during the study period, olanzapine was the principal PO antipsychotic used. Our findings may not be extrapolatable to other PO second-generation antipsychotics, such as risperidone^56^ or quetiapine.^58^

## Conclusions

In this quality improvement study, emergency physicians successfully adopted the use of oral medications to treat agitation in the ED, which was primarily due to intoxication. We did not detect a difference in time to adequate sedation when PO or IM medications were used as primary therapy. These data support best-practice guidelines that suggest oral medications should be first-line treatment for agitation in the ED.

## Notes

Supplement 1. **eTable 1.** Established Behavioral Scoring Systems **eTable 2.** Classifications of Diagnoses by International Statistical Classification of Diseases and Related Health Problems, Tenth Revision (ICD-10) Codes **eTable 3.** MIAHTAPS Violence Risk Assessment Tool **eTable 4.** Patient Characteristics From 2018 to 2024 Among Patients at Risk for Agitation **eTable 5.** Additional Characteristics of Patients With Agitation During the Prospective QI Intervention From 2020 to 2021 in the Locked Intoxication Observation Unit **eTable 6.** De-Escalation Use Among Patients With Agitation During the Prospective QI Intervention From 2020 to 2021 Gathered in the Locked Intoxication Observation Unit **eTable 7.** Additional Medication Details and Outcomes Among Patients With Agitation Who Received a Sedating Medication During the Prospective QI Intervention From 2020 to 2021 Gathered in the Locked Intoxication Observation Unit **eTable 8.** Time to Adequate Sedation by Commonly Administered Medications Among Patients With Agitation Who Received a Sedating Medication During the Prospective QI Intervention Gathered From the Locked Intoxication Observation Unit **eTable 9.** Medication Details and Outcomes Among Patients With Severe Agitation (Defined as AMSS ≥2) Who Received a Sedating Medication During the Prospective QI Intervention From 2020 to 2021 in the Locked Intoxication Observation Unit

Supplement 2.
Data Sharing Statement
