# Supplementary material for: Oral Medications for Treating Agitation in a Safety Net Emergency Department
Source: JAMA Netw Open. 2025 Dec 30;8(12):e2551683. doi: 10.1001/jamanetworkopen.2025.51683 (PMC12754681; doi:10.1001/jamanetworkopen.2025.51683)
Supplement: Supplement 1. — eTable 1. Established Behavioral Scoring Systems eTable 2. Classifications of Diagnoses by International Statistical Classification of Diseases and Related Health Problems, Tenth Revision (ICD-10) Codes eTable 3. MIAHTAPS Violence Risk Assessment Tool eTable 4. Patient Characteristics From 2018 to 2024 Among Patients at Risk for Agitation eTable 5. Additional Characteristics of Patients With Agitation During the Prospective QI Intervention From 2020 to 2021 in the Locked Intoxication Observation Unit eTable 6. De-Escalation Use Among Patients With Agitation During the Prospective QI Intervention From 2020 to 2021 Gathered in the Locked Intoxication Observation Unit eTable 7. Additional Medication Details and Outcomes Among Patients With Agitation Who Received a Sedating Medication During the Prospective QI Intervention From 2020 to 2021 Gathered in the Locked Intoxication Observation Unit eTable 8. Time to Adequate Sedation by Commonly Administered Medications Among Patients With Agitation Who Received a Sedating Medication During the Prospective QI Intervention Gathered From the Locked Intoxication Observation Unit eTable 9. Medication Details and Outcomes Among Patients With Severe Agitation (Defined as AMSS ≥2) Who Received a Sedating Medication During the Prospective QI Intervention From 2020 to 2021 in the Locked Intoxication Observation Unit [file jamanetwopen-e2551683-s001.pdf]

Supplementary Online Content

Cole JB, Hurreh KM, Taghizadeh LA, et al. Oral medications for treating agitation in a safety net emergency department. *JAMA Netw Open*. 2025;8(12):e2551683.
doi:10.1001/jamanetworkopen.2025.51683

eTable 1. Established Behavioral Scoring Systems
eTable 2. Classifications of Diagnoses by *International Statistical Classification of Diseases and Related Health Problems, Tenth Revision (ICD-10)* Codes
eTable 3. MIAHTAPS Violence Risk Assessment Tool
eTable 4. Patient Characteristics From 2018 to 2024 Among Patients at Risk for Agitation
eTable 5. Additional Characteristics of Patients With Agitation During the Prospective QI Intervention From 2020 to 2021 in the Locked Intoxication Observation Unit
eTable 6. De-Escalation Use Among Patients With Agitation During the Prospective QI Intervention From 2020 to 2021 Gathered in the Locked Intoxication Observation Unit
eTable 7. Additional Medication Details and Outcomes Among Patients With Agitation Who Received a Sedating Medication During the Prospective QI Intervention From 2020 to 2021 Gathered in the Locked Intoxication Observation Unit
eTable 8. Time to Adequate Sedation by Commonly Administered Medications Among Patients With Agitation Who Received a Sedating Medication During the Prospective QI Intervention Gathered From the Locked Intoxication Observation Unit
eTable 9. Medication Details and Outcomes Among Patients With Severe Agitation (Defined as AMSS ≥2) Who Received a Sedating Medication During the Prospective QI Intervention From 2020 to 2021 in the Locked Intoxication Observation Unit

This supplementary material has been provided by the authors to give readers additional information about their work.

© 2025 Cole JB et al. *JAMA Network Open*.

eTable 1: Established Behavioral Scoring Systems

Altered Mental Status Scale (AMSS)				
Score	Responsiveness	Speech	Facial Expression	Eyes
+4	Combative, very violent, or out of control	Loud outbursts	Agitated	Normal
+3	Very anxious, agitated, mild physical element of violence	Loud outbursts	Agitated	Normal
+2	Anxious, agitated	Loud outbursts	Normal	Normal
+1	Anxious, restless	Normal	Normal	Normal
0	Responds readily to name in normal tone	Normal	Normal	Clear, no ptosis
-1	Lethargic response to name	Mild slowing or thickening	Mild relaxation	Glazed or mild ptosis (< half eye)
-2	Responds only if name is called loudly	Slurring or prominent slowing	Marked relaxation (slacked jaw)	Glazed and marked ptosis (> half eye)
-3	Responds only after minor prodding	Few recognizable words	Marked relaxation (slacked jaw)	Glazed and marked ptosis (> half eye)
-4	Does not respond to mild prodding	Few recognizable words	Marked relaxation (slacked jaw)	Glazed and marked ptosis (> half eye)

© 2025 Cole JB et al. *JAMA Network Open*.

	or shaking			

**Richmond Agitation-Sedation Scale**		
Score	Term	Description
+4	Combative	Overtly combative or violent; immediate danger to staff
+3	Very agitated	Pulls on or removes tube(s) or catheter(s) or has aggressive behavior toward staff
+2	Agitated	Frequent nonpurposeful movement or patient-ventilator dyssynchrony
+1	Restless	Anxious or apprehensive but movements not aggressive or vigorous
0	Alert and calm	
-1	Drowsy	Not fully alert, but has sustained (more than 10 seconds) awakening, with eye contact, to voice
-2	Light sedation	Briefly (less than 10 seconds) awakens with eye contact to voice
-3	Moderate sedation	Any movement (but no eye contact) to voice
-4	Deep sedation	No response to voice, but any movement to physical stimulation
-5	Unarousable	No response to voice or physical stimulation

**Behavioral Activity Rating Scale (BARS)**

© 2025 Cole JB et al. *JAMA Network Open*.

Score	Descriptor
1	Difficult or unable to rouse
2	Asleep but responds normally to verbal or physical contact
3	Drowsy, appears sedated
4	Quiet and awake (normal level of activity)
5	Signs of overt (physical or verbal) activity, calms down with instructions
6	Extremely or continuously active, not requiring restraint
7	Violent, requires restraint

© 2025 Cole JB et al. *JAMA Network Open*.

eTable 2: Classifications of Diagnoses by *International Statistical Classification of Diseases and Related Health Problems, Tenth Revision (ICD-10)* Codes

Agitation diagnosis ICD-10 code: R45.1
Alcohol intoxication diagnoses ICD-10 codes: F10.10, F10.12, F10.20, F10.22, F10.90, F10.92, R41.82, R40.
Drug intoxication diagnoses ICD-10 codes: F11.12, F11.22, F11.92, T40.0, T40.1, T40.2, T40.3, T40.4, F14, F15, T43.62, T44.9.
Psychiatric diagnoses ICD-10 codes (bipolar disorder or psychosis): F20, F21, F22, F23, F24, F25, F26, F27, F28, F29, F31.

© 2025 Cole JB et al. *JAMA Network Open*.

eTable 3: MIAHTAPS Violence Risk Assessment Tool

Behavior Exhibited	Descriptions	Score (sum each category, maximum = 12)**
Altered **M**ental Status	Appears confused, disoriented, disorganized, intoxicated with drugs or alcohol	Yes = 1, No = 0
**I**rritable	Upset, easily startled, easily annoyed, alarmed, mumbling, but cooperative	Yes = 1, No = 0
**A**gitated	Rapid Speech, hyperventilation, uncooperative, raised voice/shouting, flailing around in bed	Yes = 1, No = 0
**H**istory of Violence Against Others	Any history of violence against others (chart review)*	Yes = 2, No = 0
**T**hreatening Verbal/Physical	Physical/verbal actions with clear intent to intimidate or cause harm	Yes = 3, No = 0
**A**ttacking Objects	A physical attack directed at an object, *not* a person	Yes = 3, No = 0
**P**acing and/or **S**taring	Not breaking eye contact, prolonged glaring, pacing	Yes = 1, No = 0
* History of violence assessed by keyword searching the patient's electronic medical record for "violent" or "violence" and by reviewing the "FYI" tab of our electronic medical record (Epic, Verona, WI) for past episodes of violence.		
** MIAHTAPS scores are summed; a score of 0-2 translates to low-medium risk, a score of 3 or 4 translates to high risk (3-4), and scores ≥ 5 translate to a severe (highest) risk for violence		

© 2025 Cole JB et al. *JAMA Network Open*.

MIAHTAPS Score Ranges and Corresponding Interventions		
**Risk Score 0-2 (Low to Medium Risk)**	**Risk Score 3-4 (High Risk)**	**Risk Score ≥5 (Severe Risk)**
• No interventions needed	• Contact provider to consider as needed (PRN) medications	• Give PRN medication per RN assessment
• Comfort/sensory interventions as needed, such as:	• Indicate risk status to other caregivers	• Indicate risk status to other caregivers
• Warm blanket	• Orient patient to plan of care	• Provide safe environment
• Activities (e.g., coloring books, word or number games, etc. as appropriate)	• Allow patient time and space to vent	• Guide staff away as needed for safety
• Nourishment PRN	• Offer PRN medications	• Notify security
	• Comfort/sensory interventions (see list under low to medium risk category)	• Consider restraints
		• Request provider to bedside
		• Comfort/sensory interventions (see list under low to medium risk category)

© 2025 Cole JB et al. *JAMA Network Open*.

eTable 4: Patient characteristics from 2018 to 2024 among patients at risk for agitation[a]

Characteristic	Pre-intervention<br>*n* = 35,483	Post-intervention<br>*n* = 53,761
Age, median (IQR), years	40 (30-54)	39 (30-54)
Female sex	9,130 (26)	14,361 (27)
Race/Ethnicity[b]		
American Indian or Alaska Native	5,351 (15)	6,949 (13)
Asian	475 (1)	831 (2)
Black, non-Hispanic	12,598 (36)	21,232 (40)
White, non-Hispanic	14,384 (41)	19,844 (37)
Hispanic	1,949 (6)	3,696 (7)
Native Hawaiian or Pacific Islander	72 (0.2)	179 (0.3)
Unknown or patient declined	654 (2)	1,030 (2)
Mode of arrival		
Emergency medical services	23,042 (65)	38,550 (72)
Police	3,973 (11)	3,703 (7)
Walk-in	8,468 (24)	11,508 (21)
Breath or blood alcohol > 0	20,400 (58)<br>[*n* = 24,626]	24,301 (45)<br>[*n* = 36,082]
Alcohol concentration if > 0, median (IQR), %	0.21 (0.15-0.27)	0.21 (0.14-0.27)
Disposition		
Discharged	29,089 (82)	45,907 (85)
Admitted to the hospital	6,348 (18)	7,735 (14)
Left before discharge	46 (0.1)	119 (0.2)
Diagnosis		
Agitation	3,512 (10)	2,573 (5)
Alcohol intoxication	30,512 (86)	43,008 (80)

© 2025 Cole JB et al. *JAMA Network Open*.

Drug intoxication	2,890 (8)	5,815 (11)
Bipolar or psychosis	1,420 (4)	2,373 (4)
Sedating medication received, any	11,720 (33)	15,546 (29)
Route of first sedating medication		
Intramuscular or intravenous	11,244/11,720 (96)	10,085/15,546 (65)
Intramuscular	7,692/11,720 (66)	4,913/15,546 (32)
Intravenous	3,552/11,720 (30)	5,172/15,546 (33)
Oral	476/11,720 (4)	5,461/15,546 (35)

Data presented as *n* (%) or median (IQR)

a. Including patients roomed in a locked unit and patients diagnosed with alcohol intoxication, drug intoxication, agitation, bipolar mood disorder, or psychosis.
b. More than one race was listed for some patients, thus the total exceeds the number of patients

© 2025 Cole JB et al. *JAMA Network Open*.

eTable 5: Additional characteristics of patients with agitation in the locked intoxication observation unit during the prospective QI intervention from 2020 to 2021.

**Characteristic**	**No sedation (*n* = 508)**	**Oral sedation (*n* = 446)**	**Intramuscular sedation (*n* = 732)**
Comorbidities			
Hypertension	107 (21)	80 (18)	114 (16)
Diabetes	29 (6)	16 (4)	34 (5)
Liver Disease or cirrhosis	21 (4)	18 (4)	30 (4)
Human immunodeficiency virus	7 (1)	4 (1)	15 (2)
Obstructive lung disease	64 (13)	49 (11)	71 (10)
Coronary Artery Disease	8 (2)	6 (1)	4 (1)
Schizophrenia	40 (8)	32 (7)	39 (5)
Bipolar disorder	35 (7)	42 (9)	57 (8)
Intravenous drug use	18 (4)	23 (5)	27 (4)
No comorbidities	284 (56)	249 (56)	487 (67)
Vital signs on arrival, median (IQR)			
Temperature, °Celsius	36.6 (36.4-36.8)	36.6 (36.4-36.8)	36.6 (36.3-36.8)
Heart rate, beats per min	91 (80-103)	97 (87-112)	91 (81 -104)
Respiratory rate, per min	18 (16-18)	18 (16-19)	18 (16-18)
Systolic blood pressure, mmHg	126 (114-140)	128 (115-142)	125 (112-139)
Oxygen saturation, %	97 (95-99)	97 (96-99)	97 (95-99)
Reason no de-escalation attempted[a]			
Not possible due to violence	0/15	1/8 (13)	35/62 (56)
Not possible due to extreme agitation	0/15	2/8 (25)	55/62(89)
Verbal threats of violence	0/15	5/8 (63)	13/62 (21)
EMS reported significant history of violence	1/15 (7)	0/8	12/62 (19)

© 2025 Cole JB et al. *JAMA Network Open*.

Unknown	14/15 (93)	0/8	0/62

Data presented as *n* (%) or median (IQR). EMS, emergency medical services. The oral sedation and intramuscular sedation groups refer to patients who received an oral or intramuscular sedating medication, respectively, as the first sedating medication.

a. Some patients had more than one reason that de-escalation was not attempted

© 2025 Cole JB et al. *JAMA Network Open*.

eTable 6: De-escalation use among patients with agitation in the locked intoxication observation unit during the prospective QI intervention from 2020 to 2021.

Intervention Observed	No sedation (*n* = 508)	Oral sedation (*n* = 446)	Intramuscular sedation (*n* = 732)
De-escalation attempted for agitation	493 (97)	438 (98)	670 (92)
Offering nourishment (juice, crackers, meal)	121 (24)	113 (25)	108 (15)
Offering an object of comfort (pillow, warm blanket)	132 (26)	110 (25)	160 (22)
Period of intent listening (at least 20 seconds)	285 (56)	236 (53)	343 (47)
Identifying the patients' wants or needs (e.g. "what would you find helpful?"	128 (25)	109 (24)	139 (19)
Offering choices or options (e.g. "Would you like to try a medication to help you relax?"	88 (17)	242 (54)	193 (26)
Calmly providing encouragement to modify behavior (e.g. "I think if you sat down and relaxed a bit you would feel better"	216 (43)	194 (44)	323 (44)
Offering support (e.g. "Let's figure this out together" Calling family/friends to provide update, apologizing)	85 (17)	69 (16)	86 (12)
Validating/acknowledging the situation ("I would be upset too if I were in your situation," "I understand," "I believe you," "I see this has been hard for you")	132 (26)	87 (20)	151 (21)
Physical barrier/movement (e.g. move bed to another location, bed rotation, close door)	96 (19)	73 (16)	165 (23)
Providing patient with step by step guidance of what to expect during their stay	178 (35)	118 (27)	178 (24)
Offering to let the patient use the restroom, bedpan, or urinal	80 (16)	55 (12)	105 (14)
Changing the conversation/redirecting conversation	63 (12)	43 (10)	66 (9)

Data presented as *n* (%) or median (IQR). The oral sedation and intramuscular sedation groups refer to patients who received an oral or intramuscular sedating medication, respectively, as the first sedating medication.

© 2025 Cole JB et al. *JAMA Network Open*.

eTable 7: Additional medication details and outcomes among patients with agitation in the locked intoxication observation unit who received a sedating medication during the prospective QI intervention from 2020 to 2021.

Detail	Oral sedation (*n* = 446)	Intramuscular sedation (*n* = 732)
**Offering an oral medication**		
Reason that oral medication was not offered		
Active violence	NA	205/548 (37)
Delirium or confusion	NA	169/548 (31)
Severe agitation without violence	NA	28/548 (5)
Patient not able to take anything by mouth	NA	5/548 (1)
Clinical team forgot about training	NA	2/548 (<1)
Unknown	NA	139/548 (25)
Initial patient response to oral medication[a]		
Accepted medication	434 (97)	0/184
Declined medication	4 (1)	129/184 (70)
Violence or threat of violence	2 (<1)	25/184 (14)
Verbal abuse or profanity	1 (<1)	32/184 (17)
No response or patient declined to answer	5 (1)	19/184 (10)
**Details of medication administration**		
Medication given		
Olanzapine	146 (33)	564 (77)
Olanzapine + lorazepam	263 (59)	0
Droperidol	0	129 (18)
Lorazepam	34 (8)	16 (2)
Midazolam	0	16 (2)
Risperidone	3 (1)	0

© 2025 Cole JB et al. *JAMA Network Open*.

Haloperidol	0	6 (1)
Ziprasidone	0	1 (<1)
**Additional (Rescue) Sedation**		
Need for additional sedation	142 (32)	208 (28)
Elapsed time from initial sedation to additional sedative, median (IQR), min	55 (31-97)	54 (30-122)
Route of Additional Sedation		
Intramuscular	74/142 (52)	176/208 (85)
Oral	50/142 (35)	21/208 (10)
Intravenous	18/142 (13)	11/208 (5)
**Length of Stay and Disposition**		
Length of stay in the ED, min	558 (416-782)	522 (389-712)
Disposition		
Discharged	327 (73)	564 (77)
Psychiatry evaluation	88 (20)	122 (17)
Admission to hospital floor	23 (5)	25 (3)
Admission to intensive care unit[b]	3 (1)	11 (2)
Detoxification center	4 (1)	5 (1)
Jail	1 (<1)	5 (1)
**AMSS values at the time of sedation**		
Highest AMSS[b] in 10 minutes before sedation		
0	9 (2)	2 (<1)
1	210 (47)	137 (19)
2	155 (35)	269 (37)
3	23 (5)	144 (20)
4	7 (2)	135 (18)
Unknown	42 (9)	45 (6)
AMSS at the time of medication administration		

© 2025 Cole JB et al. *JAMA Network Open*.

0	28 (6)	16 (2)
1	252 (57)	168 (23)
2	112 (25)	272 (37)
3	19 (4)	124 (17)
4	2 (<1)	117 (16)
Unknown	33 (7)	35 (5)

Data presented as *n* (%) or median (IQR). NA, not applicable. The oral sedation and intramuscular sedation groups refer to patients who received an oral or intramuscular sedating medication, respectively, as the first sedating medication.

a. This variable lists the patient's initial response. Some patients changed their mind after further de-escalation. Some patients had more than one response to the offer of oral medication.
b. This includes one patient and five patients in the oral and intramuscular groups, respectively, who underwent tracheal intubation in the ED

© 2025 Cole JB et al. *JAMA Network Open*.

eTable 8: Time to adequate sedation by commonly administered medications among patients with agitation in the locked intoxication observation unit who received a sedating medication during the prospective QI intervention from 2020 to 2021.

**Medication outcome**	**Oral sedation (*n* = 446)**	**Intramuscular sedation (*n* = 732)**
**Initial Sedation Medication**		
**Olanzapine**	146 (33)	564 (77)
Time to adequate sedation, median (IQR), min	15 (9-30)	15 (9-27)
**Olanzapine + lorazepam**	263 (59)	0
Time to adequate sedation, median (IQR), min	15 (8-37)	N/A
**Droperidol**	0	129 (18)
Time to adequate sedation, median (IQR), min	N/A	14 (9-21)

* data presented as *n* (%) or median (IQR). The oral sedation and intramuscular sedation groups refer to patients who received an oral or intramuscular sedating medication, respectively, as the first sedating medication.

© 2025 Cole JB et al. *JAMA Network Open*.

eTable 9: Medication details and outcomes among patients in the locked intoxication observation unit with severe agitation (defined as AMSS ≥+2[a]) who received a sedating medication during the prospective QI intervention from 2020 to 2021.

**Medication details and events**	**Oral sedation (*n* = 206)**	**Intramuscular sedation (*n* = 572)**	**Difference**
Offered oral medication	206 (100)	138 (24)	76% (72%-79%)
**Details of medication administration**			
Time from ED arrival to sedation administration, median (IQR), min	32 (18-52)	21 (11-52)	6 (3-10)
Medication given			
Olanzapine	70 (34)	441 (77)	Not calculated
Olanzapine + lorazepam	121 (59)	0	Not calculated
Droperidol	0	107 (19)	Not calculated
Lorazepam	14 (7)	7 (1)	Not calculated
Midazolam	0	12 (2)	Not calculated
Risperidone	1 (<1)	0	Not calculated
Haloperidol	0	4 (1)	Not calculated
Ziprasidone	0	1 (<1)	Not calculated
**Effectiveness Outcomes**			
Time to adequate sedation, median (IQR), min[b]	16 (10-34)	16 (9-28)	2 (0 to 4)
Need for additional sedating medication	74 (36)	172 (30)	6% (-2% to 13%)
Elapsed time from initial sedating medication to additional medication, median (IQR), min	55 (30-97) [n=74]	49 (28-122) [n=172]	1 (-12 to 14)
**Violent acts and verbal abuse towards healthcare workers[c]**			

© 2025 Cole JB et al. *JAMA Network Open*.

Violent act, attempted or inflicted	13 (6)	92 (16)	Not calculated
Threat of bodily harm	42 (20)	193 (34)	Not calculated
Verbal abuse	80 (39)	302 (53)	Not calculated

Data presented as *n* (%) or median (IQR). The oral sedation and intramuscular sedation groups refer to patients who received an oral or intramuscular sedating medication, respectively, as the first sedating medication.

a. The AMSS is an ordinal agitation scale from -4 (unresponsive) to 0 (normal) to +4 (most agitated)

© 2025 Cole JB et al. *JAMA Network Open*.
