# Supplementary material for: Oral Medications for Treating Agitation in a Safety Net Emergency Department
Source: JAMA Netw Open. 2025 Dec 30;8(12):e2551683. doi: 10.1001/jamanetworkopen.2025.51683 (PMC12754681; doi:10.1001/jamanetworkopen.2025.51683)
Supplement: Supplement 2. — Data Sharing Statement [file jamanetwopen-e2551683-s002.pdf]

# Data Sharing Statement

Cole. Oral Medications for Treating Agitation in a Safety Net Emergency Department. *JAMA Netw Open*. Published December 30, 2025. doi:10.1001/jamanetworkopen.2025.51683

**Data**
**Data available:** Yes
**Data types:** Deidentified participant data
**How to access data:** Please email brian.driver@hcmed.org for data requests.
**When available:** With publication

**Supporting Documents**
**Document types:** None

**Additional Information**
**Who can access the data:** The data will be made available to researchers whose proposed use of the data has been approved.
**Types of analyses:** The data will be made available on a case by case basis based on the type of proposal.
**Mechanisms of data availability:** Data will be made available with investigator support, after approval of a proposal, and after completion of a signed data use agreement.
